# A-Book: A Feedback-Based Adaptive System to Enhance Meta-Cognitive Skills during Reading

**DOI:** 10.3389/fnhum.2017.00098

**Published:** 2017-03-13

**Authors:** Ernesto Guerra, Guido Mellado

**Affiliations:** ^1^Experimental Psycholinguistics Lab, Pontificia Universidad Católica de ChileVillarrica, Chile; ^2^Center for Intercultural and Indigenous ResearchVillarrica, Chile

**Keywords:** adaptive ICTs, meta-cognitive skills, reading comprehension

## Abstract

In the digital era, tech devices (hardware and software) are increasingly within hand’s reach. Yet, implementing information and communication technologies for educational contexts that have robust and long-lasting effects on student learning outcomes is still a challenge. We propose that any such system must a) be theoretically motivated and designed to tackle specific cognitive skills (e.g., inference making) supporting a given cognitive task (e.g., reading comprehension) and b) must be able to identify and adapt to the user’s profile. In the present study, we implemented a feedback-based adaptive system called A-book (assisted-reading book) and tested it in a sample of 4th, 5th, and 6th graders. To assess our hypotheses, we contrasted three experimental assisted-reading conditions; one that supported meta-cognitive skills and adapted to the user profile (adaptive condition), one that supported meta-cognitive skills but did not adapt to the user profile (training condition) and a control condition. The results provide initial support for our proposal; participants in the adaptive condition improved their accuracy scores on inference making questions over time, outperforming both the training and control groups. There was no evidence, however, of significant improvements on other tested meta-cognitive skills (i.e., text structure knowledge, comprehension monitoring). We discussed the practical implications of using the A-book for the enhancement of meta-cognitive skills in school contexts, as well as its current limitations and future developments that could improve the system.

## Introduction

The advent of increasingly accessible and cheaper digital information and communication technologies (ICTs) has raised the question about their role in the context of formal education. ICTs are seen by some as a natural, intuitive, and easy-to-use tool for mediated learning, and there are some examples of their successful application in education (e.g., Roschelle et al., 2000; Thibaut et al., 2015). There are, nevertheless, detractors and critics of indiscriminate use of ICTs in school contexts (e.g., Buckingham, 2007) and also less successful examples of their application to classrooms (see, e.g., Kramarski and Feldman, 2000). Currently, it is still unclear what kind of technology is most appropriate to support children’s learning and development (see Hermans et al., 2008). What is clear, however, is that only making ICTs available to schools does not guarantee a significant impact in student performance and learning processes (Cuban et al., 2001; Selwyn et al., 2009). The answer to this question might depend partly on whether the technology in use is specifically designed to tackle relevant cognitive processes supporting specific capabilities (see, e.g., Blok et al., 2002).

In the context of language education, ICTs have been predominately used as an aid for low-level language skills, such as decoding (e.g., Barker and Torgesen, 1995; Mathes et al., 2001; Bonacina et al., 2015) and less to help students to improve text comprehension skills (see National Reading Panel, 2000). It is well-established that word decoding is critical for reading comprehension during primary school years (e.g., Perfetti and Hogaboam, 1975; Kendeou et al., 2009). However, it is also known that meta-cognitive strategies such as inference making, comprehension monitoring, and text structure knowledge are relevant for understanding written stories, in particular when children transition from learning to read to learning by reading (see Paris et al., 1983; Oakhill et al., 2014). How could new technologies be used most effectively to foster and enhance these skills?

A number of reviews suggest that there is virtually no evidence of the benefits that ICT could provide to reading comprehension during school years (see Torgerson and Elbourne, 2002; See and Gorard, 2014; Paul and Clarke, 2016). An existing study examined the effects of the use of a software that focused on reading and spelling (Brooks et al., 2006). The authors report that the software allowed students to hear and correct themselves and work independently at their pace and had different difficulty levels to which students were assigned based on prior assessment. Pre- and post-treatment test were administered to the experimental and the control group. Children in the ICT group undertook sessions of 1 h a day for 10 consecutive school days. Statistical comparison showed an advantage in the reading test for the control group compared to the ICT group, suggesting a negative effect of the use of the software. Similarly, a study by Khan and Gorard () assessed the effectiveness of a computer program designed to improve reading. The ICT consisted in a multi-sensory software that combined touch, vision and sound; it provided more than 100 texts, immediate feedback and the difficulty of items could be adapted. User’s progress was also recorded by the software. In the study, participant’s literacy skills were assessed pre- and post-treatment. Student in the ICT condition used the software for 10 weeks, period over which the control group did not use the software. Statistical analysis showed that both the control and the ICT group improved literacy skills after the 10 weeks period. Again, however, the control group performed significantly better than the ICT group.

Other studies have also failed to demonstrate the usability of ICT (see Rouse and Krueger, 2004; Lei and Zhao, 2007; Given et al., 2008; Borman et al., 2009). In contrast, interventions delivered to groups of students directly by teachers seem to be much more successful in improving participants reading skills (see Berkeley et al., 2011; Vaughn et al., 2011; McMaster et al., 2012). In this context, it might be tempting to argue that ICT are not a suitable tool to foster and improve reading comprehension. An alternative view is that ICT must be designed and grounded theoretically, must tackle specific cognitive skills supporting reading comprehension (rather than providing an “enhanced” reading experience, see Khan and Gorard, 2012), and in addition, they should be able to adapt online to the individual characteristics and performance of the student (see McMaster et al., 2012).

In this article, we present the implementation and a preliminary assessment of an automatized feedback-based system we called A-book (assisted-reading book), designed to provide theoretically motivated user-based feedback during the process of reading. The A-book’s aim is to offer an adequate context for primary school students to develop meta-cognitive strategies at an early stage. In a between-subject design we contrasted three experimental assisted-reading conditions; a training condition, an adaptive condition and a control condition. In all three experimental conditions, readers were presented with stories (one page at the time) and three types of *yes*-or-*no* questions. Each of these questions related to a critical meta-cognitive ability, namely, inference making, comprehension monitoring and text structure knowledge. Our working hypothesis was that pertinent feedback (on inference, monitoring, and structure) should prompt the young reader to begin to strategically apply these skills while reading (i.e., training and adaptive conditions) in particular when the system adapts to the users’ profile purposefully focusing on meliorating her weaknesses (i.e., adaptive condition). Consequently, we predicted that both the training and adaptive conditions would in time produce better comprehension accuracy scores relative to the control condition. Furthermore, we hypothesized that the adaptive behavior of the system should benefit the user comprehension processes. Thus, children using the adaptive condition of the system should outperform the training condition group.

## Materials and Methods

### Participants

Ninety primary school students from 4th, 5th, and 6th grade (aged between 9 and 12 years) from a local school, who participated voluntarily on a session basis, were recruited to take part of the study. All children were monolingual Spanish native speakers.

### Materials

#### Reading Materials

We selected 10 stories from “Un cuento al día – Antología” (Consejo Nacional del Libro y la Lectura, 2013), a book published by the Consejo Nacional de la Cultura y las Artes (National Board for Culture and the Arts), Government of Chile. This book was made freely available in 2013, in the context of the Plan Nacional de Fomento de la Lectura (National Plan for the Reinforcement of Reading) “Lee Chile Lee,” and it was aimed to promote parental reading as a daily activity^1^. Each story was divided in a number of fragments (*m* = 12.7; range: 9–27) of about 100 words each (*m* = 124; range: 70–200) for their presentation. For each fragment, we wrote three questions, each of them related to a specific meta-cognitive skill (i.e., inference making, comprehension monitoring and text structure knowledge; see the Supplementary Material for some examples). For each question, we then wrote two kinds of feedback (i.e., explanatory and control) and each of them in two equivalent versions (one for correct answers and one for incorrect answers).

Questions aimed to capture the readers’ ability to make inferences were constructed to ask about information that was not explicitly given in the text. For instance, if the text said ‘the old man mixed the content of jars to make rain…,’ we asked whether ‘the old man knew a recipe for rain.’ The explanatory feedback alluded to such critical information, by stating for instance, ‘if the old man mixed the content of jars to make rain, he most probably knew a recipe for rain.’ Questions about the structure of the text directly asked the reader whether the story’s characters had been already presented, whether they already knew the scenario or context in which the story was taken place, or whether the story was about to end, or only at the beginning. Such questions did not include any content of stories, in other words they were story independent. The corresponding feedback was also story independent insofar it just reminded the reader the linkage between characters, scenario, story conflict and conflict resolution, and the structure of the text (e.g., ‘Exactly, if you are beginning to know the characters, the story is just starting’; ‘Hmmm, I am not sure… if you are beginning to know the characters, the story is just starting’).

Finally, questions intended to measure participants’ comprehension monitoring, were always built as ‘Did you realized that…’ and the sentence was completed by a literal phrase of the text or one slightly modified for *No*-answers. Feedback for correct *Yes*-answers consisted on a reinforcement sentence that referred to the concentration of the reader (‘Very good! It is clear that you are very concentrated’), while the feedback for correct answers *No*-answers always began by saying: ‘Of course not, because that never happened.’ and ended with the reinforcement sentence. When the readers responded incorrectly, the explanatory feedback consisted on a re-iteration of the text cited from the text plus the mentioning of the importance of paying attention to what one understands, as well as to what one does not clearly understand.

#### Assisted-Reading Experimental Conditions

Our study contrasted three assisted-reading experimental conditions, two of them with explanatory feedback (i.e., training and adaptive experimental conditions) and one control condition. In the training and adaptive experimental conditions, the feedback the readers received after responding a question was aimed to encourage them to reflect and think over the question and the answer they chose, independently of whether the given answer was correct or not. Thus, the training/adaptive feedback provided the reader with an explanation for the answer of the question, while at the same time reassured readers when answering correctly (e.g., “Very good…,” “Well done...”) and inviting reconsideration (e.g., “Hmm, I am not so sure, perhaps… […] Don’t you think?”), when the response was incorrect (instead of penalizing it). The logic behind inviting reconsideration was to keep both training- and adaptive-feedback explanatory in nature. In other words, the feedback should point to the relevant information necessary to answer the question accurately, independently of whether the actual answer of the participant was correct or incorrect.

The critical difference between the training and adaptive assisted-reading experimental conditions was the way in which the selection of the meta-cognitive skill, in other words the type of question (i.e., inference making, story structure, or comprehension monitoring), was presented to the reader in a particular moment. Presentation of questions in the training condition was counterbalanced: for each story, participants read the same number of questions on each meta-cognitive skill and their presentation was pseudo-randomized. The adaptive experimental condition, instead, selected the weakest meta-cognitive skill at the user level and prioritized its presentation. For this reason, the adaptive experimental condition required an individual profile as a starting point to work, and such data was obtained from participants’ session using the training condition (see Design). It also joined text fragments, first two, then three, for readers with accuracy higher than 75% (two fragments) and 85% (three fragments) in all meta-cognitive skills. The control condition also presented counterbalanced question types but the feedback consisted only in the word “Correct” or “Incorrect,” depending on whether the answer given by the reader was correct or not.

### Design

We constructed a website we used for presentation and management of the stories and data. All the stories, questions and feedback were presented one at the time on the screen. In such way, the readers could concentrate on a single task at the time (e.g., reading text, answering the question, reading feedback). Participants were given six 30-min reading sessions in two blocks (three sessions on each block) over a 2 weeks period (see **Table 1**). The first three sessions (week 1) formed the Exposure Block, while the subsequent three sessions (week 2) constituted the Testing Block. In the first block (week 1), the full sample of participants was randomly divided into two groups; one with approximately twice as many participants as the other (*n* = 34 and *n* = 58). The smaller group was assigned to the control condition, while the larger group was assigned to the training condition. During this block, participants were presented with five stories, each of which had nine fragments to be read, and 27 questions to be answer (nine on each experimental condition, i.e., inference making, text structure, and comprehension monitoring).

**Table 1:** Design of the study.

Week 1 (Exposure)			Week 2 (Assessment)		
Session 1	Session 2	Session 3	Session 4	Session 5	Session 6
10–15 participants per session			10–15 participants per session		
Experimental conditions: Training | Control			Experimental conditions: Training | Control | Adaptive		

The exposure session was aimed to familiarized participants with the task before comparing the effects of different reading conditions. In this sense, participants should have an experience of continuity between the two blocks. Thus, we presented the control condition to a third of the participants and the training condition to the other two thirds; participants in the control condition worked on that condition across blocks. Moving from the training to the adaptive should be unnoticed; in fact, participants would just have more questions of that task that is difficult for them. In contrast, if we would have for instance presented everyone with the control condition in the exposure block, the second block would have meant a different context for those participants in the training and adaptive conditions. In our view, this could have created a disadvantage for those conditions.

In the second block, 30 of the participants in the training condition stayed on that condition, while the other 28 were assigned to the adaptive condition. This design allowed the correct functioning of the adaptive condition, feeding readers’ data in the second block from the data collected at the participant level during the first block in the training condition. In the second block, participants were presented with five new stories. The number of fragment per stories varied between 9 and 27 and for each fragment there were always three potential questions corresponding to each meta-cognitive skill.

### Procedure

**FIGURE 1:**
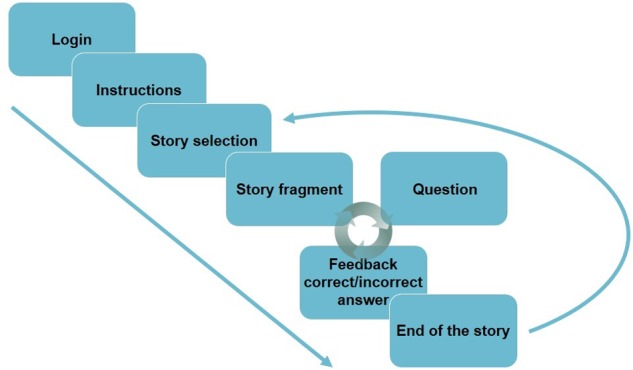
**A schematic representation of the reading task**.

On each reading session, the students were invited to participate voluntarily. No personal data from students was recorded and all of them participated voluntarily on a session basis. **Figure 1** shows a schematic presentation of the sequence that participants would see when performing the task. Every student was assigned a username (previously created) to enter the website and work individually on a computer. The number of participants per session varied between 10 and 15 students at the time. As participants logged in the website, they were first presented with written instructions. In these instructions, they were informed that they would read the stories fragment by fragment and receive questions about them. It also included words of encouragement such as ‘We want to invite you to read… but we want your reading to be as fun as possible.’ After reading the instructions, participants clicked on a ‘Next’-button and were presented with five pictures, each of them representing one story.

Students could read the titles and select any out of five stories to read. When they clicked on a picture they were presented with the title and first fragment of the story they selected. There was no time constraint and participants could read the story fragments at their own pace. When participants clicked on a ‘Next’-button, they were presented with a written question, plus a ‘Yes’- and a ‘No’-button. After they gave their response, the response feedback was presented on the screen plus a ‘Next’-button. When they pressed ‘Next’ a new fragment was presented, and the loop of fragment-question-feedback continued until the end of the story. When the story ended, participants read a message stating that they had finished the story and encouraged them to read other stories or even the same one again if they wanted. However, the icon of the most recently read story temporally disappeared and did appear again only after a different story was fully read. When an already-read story was chosen by the participant, the system would assign a new question for each fragment until there were no more questions to be answered. This meant that each story could be fully read up to three times, after which the icon for the story disappeared from the story selection screen.

The procedure was approved by the Ethics Committee of the Campus Villarrica. All activities were performed in the school dependencies and during regular school hours as a complementary informatics and language activity. The participation of the students was approved by the Principal of the school and the head of the Technical Pedagogical Unit as the legally authorized representatives.

### Data analysis

Before inferential analysis, we examined individual participants’ responses and decided to exclude four participants since they gave only ‘Yes’ responses. All other data were included in the analysis. Our basic dependent variable was participants’ accuracy, but we were also interested in seeing how such accuracy developed in time and, particularly, as a function of the different assisted-reading experimental conditions. One clear candidate variable to evaluate such effect in time was the number of responses at the participants’ level. The more questions they responded, the more experience they are supposed to gain. If there are any differences in the effect of such experience on participants’ accuracy as a function of the assisted-reading experimental condition, we should observe them by contrasting the three experimental conditions across time (as reflected by the number of responses). However, due to the nature of the task, data were strongly unbalanced in the number of responses per participant, per condition and per meta-cognitive skill. The adaptive condition exhibits much less questions of text structure relative to inferences and comprehension monitoring, while the control condition exhibits overall much less questions per participant (maximum number of questions = 142, compared to 208 and 243 for training and adaptive conditions, respectively).

Consequently, we decided to group the number of questions based on four quartiles per experimental condition and meta-cognitive skill. In doing so, we found a principled way to obtain a more balanced data set for comparison. **Table 2** shows the cutting values that divided the percentile groups per condition and skill. It also shows the number of cases per group and the cumulative percentage this number meant for the total.

**Table 2:** Descriptive statistics per experimental condition and meta-cognitive skill.

Experimental condition	Meta-cognitive skill	Percentile	Cutting value	*n*	Cumulative %
Adaptive	Inference	**25**	20	177	24
		**50**	47.5	191	50
		**75**	93	183	74.9
		**100**		185	100
		***Total***		*736*	
	Comprehension	**25**	12	146	23.6
	monitoring	**50**	28	158	49.2
		**75**	48	156	74.4
		**100**		158	100
		***Total***		*618*	
	Text structure	**25**	21	59	24.3
		**50**	51	62	49.8
		**75**	81	60	74.5
		**100**		62	100
		***Total***		*243*	

Control	Inference	**25**	15	136	23.4
		**50**	33	146	48.5
		**75**	59	151	74.4
		**100**		149	100
		***Total***		*582*	
	Comprehension	**25**	15	135	23.1
	monitoring	**50**	34.5	157	50
		**75**	59.75	146	75
		**100**		146	100
		***Total***		*584*	
	Text structure	**25**	15	145	24.9
		**50**	34	147	50.2
		**75**	59.25	145	75.1
		**100**		145	100
		***Total***		*582*	

Training	Inference	**25**	21	172	24.9
		**50**	47.5	174	50
		**75**	83	171	74.7
		**100**		175	100
		***Total***		*692*	
	Comprehension	**25**	22	170	24.8
	monitoring	**50**	47	170	49.6
		**75**	82	173	74.9
		**100**		172	100
		***Total***		*685*	
	Text structure	**25**	21	163	23.6
		**50**	47.5	183	50
		**75**	83	171	74.7
		**100**		175	100
		***Total***		*692*	

This grouping led to a more balanced data set for comparison, which we subsequently compared using a generalized linear mixed model approach, henceforth GLMM (lmerTest Package in R; see Baayen et al., 2008). GLMM are particularly suitable for the analysis of binomial data since they offer a sufficiently conservative, yet balanced approach for accuracy analysis (see Hosmer and Lemeshow, 2000; Quené and van den Bergh, 2004, 2008). GLMM allows a multilevel analysis with crossed random factors (e.g., participants) while accommodating such intrinsic variation around the fixed factors and their interaction. These models have less assumption than classic ANOVAs (do not assume homoscedasticity or sphericity of the data), do not require data aggregation and are more robust against unbalanced data and missing values (Quené and van den Bergh, 2004, 2008; Baayen et al., 2008; Barr, 2008). Their output delivers estimates, standard errors, *z*- and *p*-values.

We contrasted the effects of assisted-reading experimental conditions for each meta-cognitive skill (i.e., inference making, text structure, and comprehension monitoring). To minimize collinearity between fixed factors, we centered the predictors’ values on a mean of 0 before analysis, using a scale function (base Package in R). The models^2^ included, as fixed effects, the assisted-reading condition and the response group (quartiles) and their interaction. They also included a random intercept for participants and fixed effects and interaction random slopes for the participant random intercept. To simplify the model and improve convergence, we did not include random correlations between predictors and the random intercept (see Barr et al., 2013 for such recommendation).

## Results

**FIGURE 2:**
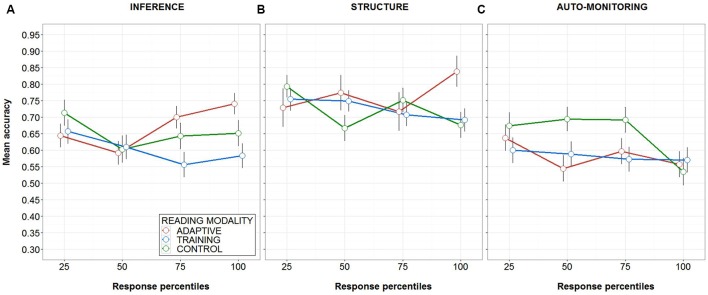
**Mean accuracy (with error bars plotting the standard error of the mean) as a function of meta-cognitive skill, response percentile and reading modality. (A–C)** correspond the results for inference making, text structure knowledge and comprehension monitoring, respectively.

The results from the GLMM for the inference experimental condition showed a main effect of condition (β = -0.17, *t* = -2.04, *p* < 0.05) but no main effect of response percentile group. More importantly, it evidenced a reliable interaction effect between the condition and response group (β = -0.15, *t* = -2.82, *p* < 0.01). **Figure 2A**, shows a graphic representation of the observed interaction pattern. Accuracy remained relatively similar for all experimental conditions within the first two quartiles, yet from the third quartile on, a distinctive pattern for each condition emerged: accuracy in the adaptive condition increased to 0.69 [CI95% ± 0.7] and then to 0.74 [CI95% ± 0.6] in the third and fourth quartile respectively, compared to the other conditions that remained at 0.64 [CI95% ± 0.8] and 0.65 [CI95% ± 0.8] for control and 0.55 [CI95% ± 0.8] and 0.58 [CI95% ± 0.8] for the training condition in the same quartiles.

In contrast to the results for the inference experimental condition, the outcome of the GLMM for the text structure experimental condition showed no main effect of condition or group, neither showed interaction between predictors (*t*-values < |2|). **Figure 2B** illustrates the mean accuracy pattern across the response groups, showing no clear differences between conditions across time. Finally, the GLMM from the comprehension monitoring experimental condition detected a main effect of response percentile (β = -0.16, *t* = -2.034, *p* < 0.05). As it is shown in **Figure 2C**, there is a tendency of a decrease in accuracy, in particular for the control condition. Such effect, however, was not modified by the condition as reflected by the absence of the predicted interaction between assisted-reading condition and the response percentile groups. **Table 3** summarizes the results of the GLMMs analysis.

**Table 3:** Main and interaction effects in the GLMM by meta-cognitive skill.

Fixed effects:	β	*SE*	*z*-value	Pr(>|z|)
**(A) Inference making**				

(Intercept)	0.628	0.080	7.871	0.000
Condition	-0.171	0.083	-2.048	0.041
Group	-0.001	0.053	-0.013	0.989
Condition^∗^group	-0.153	0.054	-2.823	0.005

**(B) Text structure**				

(Intercept)	1.052	0.092	11.418	0.000
Condition	-0.037	0.103	-0.362	0.718
Group	-0.079	0.067	-1.178	0.239
Condition^∗^group	-0.075	0.065	-1.153	0.249

**(C) Comprehension monitoring**				

(Intercept)	0.484	0.097	4.997	0.000
Condition	0.008	0.102	0.081	0.936
Group	-0.165	0.081	-2.034	0.042
Condition^∗^group	0.073	0.080	0.921	0.357

## Discussion

The current study constitutes a proof of concept for the following hypothesis: the effective use of ICTs in learning contexts depend on whether this technology is designed to enhance and support specific cognitive skills that underlie specific cognitive tasks. We proposed that any effective ICT system must be designed to provide a theoretically motivated context for learning and that such system must have the ability to adapt to the user’s profile. We chose to investigate this hypothesis in the context of text comprehension in primary school students since most studies that used ICTs in the context of language instruction either focused on basic language skills (e.g., decoding), and those that concentrated in more high-level skills did so in older readers.

Consequently, we designed and implemented a web platform that presented 4th, 5th, and 6th graders with a set of A-books, questions about them, and corresponding response feedback. Critically, we contrasted three assisted-reading experimental conditions to investigate our hypothesis, namely (1) a condition that supported specific cognitive skills (i.e., meta-cognitive abilities) and that adapted to the users’ profile (adaptive condition), (2) a condition that supported the same specific cognitive skills but did not adapt to the users’ profile (training condition), and (3) a control condition that did not support cognitive skills nor adapted to the users’ profile.

Participants read stories in one of these three different experimental conditions, while we measured their accuracy on each meta-cognitive ability question. We predicted that the adaptive condition would produce over time better accuracy scores compared to the training and the control experimental conditions, and that the training condition would surpass the results of the control condition. Indeed, the analysis of the inference experimental condition showed a reliable advantage for the adaptive condition relative to both the training and the control experimental conditions. However, the participants in the control condition performed better relative to those in the training condition (see **Figure 2A**). Moreover, analysis of the accuracy for the text structure and comprehension monitoring did not reveal such advantage for the adaptive condition. In the text structure experimental condition, we observed a late advantage for the adaptive condition (see **Figure 2B**), which could be interpreted in favor of our predictions. This advantage was nevertheless not strong enough to bring about an interaction effect between condition and response percentile group that was statistically reliable. Finally, the results observed in the comprehension monitoring experimental condition are the most puzzling ones; we observed a reliable main effect that suggests that readers’ performance deteriorated over time. The accuracy pattern, however, suggests that effect is carried by the control condition, which evidence a drop of around 15% in the fourth quartile relative to the first three quartiles.

Taken together, the present results provide support to our prediction: when readers were presented with adequate and personalized context for the support of specific cognitive skills (i.e., explanatory feedback) needed to performed specific cognitive tasks (i.e., inference making), their accuracy increased over time. Yet, there are a number of issues worth addressing with regards to the data pattern observed in the study. First, the results pattern suggests that for the inference experimental condition as for the comprehension monitoring experimental condition, readers in the control condition overcame the performance of the readers in the training condition. This unexpected result might find an explanation on the literature about students’ self-regulation and behavior modulation (Lemos, 1999; Nilson, 2013). According to Lemos, self-regulated students are better in delaying the immediate reward after a task in order to achieve more important goals. Moreover, the author suggests that self-regulation capacities are based on the assimilation of values and incentives. On the other hand, it has been suggested that the Chilean educational system teaching style is predominantly oriented to results rather than the process of learning and reflection. This, in part, as a negative consequence of the use of school rankings based on standardized evaluations as a measure of quality of education. Ortiz (2012), for example, explains that the use of these measures and rankings do not provide significant guidance for teachers to implement specific pedagogical actions, and yet among teachers (and principals) there is an increasing tendency to consider deficient results as an indicator for the need of implementation of action to improve students learning. This blind-spot (i.e., knowing that something needs to be fixed, but not knowing exactly what and how to fix it), produces many unwanted practices in schools such as the exclusion and selection of students (Ortiz, 2012; see also Flórez Petour, 2015). Interestingly, Lemos (1999) proposes that the social context can lead children to believe they are not capable to achieve expected outcomes, and that those children tend to respond maladaptatively. Instead, children that see challenges as more achievable are more likely to act more constructively. Taken together, this evidence may explain a tendency of students to respond better to short and uninformative feedback compared to the explanatory feedback. An alternative explanation, however, might be that these differences arise from the weakness of the between-subject design, view that would weaken our results. Although readers were randomly assigned to the different experimental conditions, groups were not matched in any parameter. Future research could address this issue by testing the platform in a within-subject study.

A second issue relates to the overall pattern observed for the comprehension monitoring experimental condition. The statistical analysis suggests a general tendency for a decrease in reader accuracy, in other words, the opposite to the intended effect. A potential explanation for the failure of this experimental condition could be that the questions intended to evaluate the comprehension monitoring of the comprehension process were not able to capture this meta-cognitive skill properly. Previous research has made used of the insertion of errors in the text. For instance, inconsistencies within the same paragraph were deliberately included to assess whether the reader was paying attention to the content of the story (e.g., Markman, 1979; Tunmer et al., 1983; Oakhill et al., 2005). We followed a similar logic by presenting error-free literal citation and citation with intended error. We moreover wrote the cueing phrase ‘Did you realize that…’ before each of this type of questions. However, to keep the text the same for inference making, text structure and comprehension monitoring questions, we inserted the errors after the fragment and within the question. In this context, the questions might have been too demanding and its answering logic hard to understand influencing negatively readers’ performance overtime.

This discussion leads us to a final issue of the present study. The results (in particular from the comprehension monitoring skill) rise the question on whether our intervention can produce significant changes in different meta-cognitive skills, or such improvements are limited to a specific meta-cognitive skill, in this case, the capacity of the readers to make offline inferences about the text they are reading. Our findings speak against this possibility; however, there are some attenuating points that might prompt a more optimistic view. As we argue above, the comprehension monitoring questions were most probably not the most adequate ones. Moreover, recent research has also been unsuccessful in finding improvement in comprehension monitoring both in short and long term (see Potocki et al., 2013), perhaps because this skill is much harder to foster and enhance.

With regard to the other two assessed meta-cognitive skills, participants were from the beginning less accurate on inference questions, relative to their performance on questions about the structure of the text (see **Figure 2**). This meant that they received overall much more reinforcement in the inference condition relative to text structure condition (2010 vs. 1517 questions, respectively), particularly in the adaptive condition (736 vs. 243 questions). There is nevertheless a (non-significant) trend of improvement in the last quartile in the text structure skill (see **Figure 2B**). Considering this (namely, the amount of reinforcement received) and the time readers spent using the system (only 1 week), the significant improvement observed at least for the most reinforced ability seems promising.

Without underestimating the caveats above-discussed, the results of the present study can be taken as evidence of the benefit of designing theoretically motivated (and empirically testing) ICTs interventions for educational contexts. They show that a system (and perhaps any kind of instruction) that can adapt to the user’s profile is more effective compared to those that are less flexible in the assignment of a task. Such principles are not new in the context of school teaching (e.g., Keller, 1968; Fuller, 1970), yet they have not permeated into the design and implementation of ICTs for school context (see c.f. Roschelle et al., 2000; Hammond, 2014).

### Practical Implications

One challenge for personalized teaching is avoiding the overt separation of students in different groups in the classroom or in different classrooms (see Ainscow and Miles, 2008). In this sense, the present tool allows the distinctive treatment of students in an implicit manner, that is, student do not need to be explicitly classified in groups of different achievement and being separated physically in the classroom or in different classrooms. The A-book can adapt to the user profiles even when apparently, all students are doing the same task.

**FIGURE 3:**
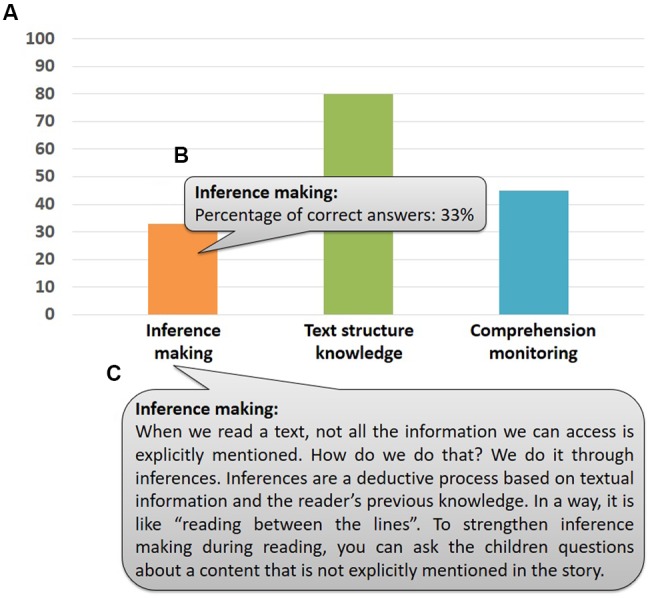
**Example of a graphical report**. The report is sent via email and presents the results as a bar plot **(A)**. When users point to a bar, the exact score for that meta-cognitive skill will pop-out **(B)**. When users point to a skill label, and explanation of it and advice for tutors will pop-out **(C)**.

Another practical implication is the potential use of the A-book as soft-assessment tool and guidance for teachers and parents. Students’ accuracy data are recorded online at the individual level. Every time a user reads a full story, an updated graphical profile is send automatically to the email address with which the user was created. In the present study, as testing phase of the system, we created all profiles prior testing and thus, we received all graphical reports. However, the basic idea is that teachers or parents create the children’s account and receive their progressive profiles. **Figure 3** shows an example of an individual report (**Figure 3A**) sent via email. This graphical information is accompanied by the exact score (by locating the mouse cursor on any bar, see **Figure 3B**) and explanations and advice (by locating the mouse cursor on any of the three meta-cognitive skills icons, see **Figure 3C**) for teachers and parents.

### Limitations and Future Directions

Indeed, the present study has limitations. Among them, we identify four, which we think can be corrected relatively easily and would mean a significant improvement for the system: first, the type of questions; second, adding more texts and of more diverse genres; third, adding a reaction time measure and minimal time for continuation; finally, making the A-book a multimodal platform. We are aware that closed-questions are not the best way to assess reading comprehension (c.f. Oakhill et al., 2014). However, we also recognize that more multifaceted questions, such as open questions, present a more complex scenario for analysis, and might demand specific training for correction. Keeping in mind that our study presents a proof of concept of an adaptive assisted-reading book that could be easily made available online, we opted for the simplest version of the answers. Knowing this is a limitation, a next step in the development of the A-book is to implement richer questions (i.e., multiple choice and content answer, true or false, completion) that can better capture the skills at stake. In connection, it seems clear that adding other text genres would allow using a more varied set of questions. Adding a larger set of texts would also allow the use of the system for a more extensive period. We observed improvement after a week, which encourages the evaluation of the A-book’s potential effect after a more prolonged usage.

Furthermore, in this first version of the A-book, we did not include a measure that could tell us how long students took to read each fragment (i.e., a reaction time measure), losing relevant data for exclusion of cases as well as for behavioral analysis. In this sense, including minimal time for continuation (e.g., calculated as 250 ms per word) would also mean an improvement. Finally, making the A-book a multimodal platform would make it not only more attractive for children and thus more likely to engage them in reading, but would also provide a much richer context, situating language within visual and auditory representations. Specifically, the insertion of illustrations accompanying text might allow the reader to construct a richer situation model of the narrative (see Arizpe and Styles, 2002 for a discussion on picturebooks), and the addition of audio-based feedback would guarantee that all students process the intended pointer and might also constitute a significant aid, in particular for less skilled readers (see Montali and Lewandowski, 1996).

## Conclusion

The present research started from the assumption that the interaction with the environment is of most relevance for the acquisition of language competencies (see Gee, 2004), without forgetting that individual differences are also critical for learning (Stanovich, 1986). Poor comprehension affects many children in primary school (Cornoldi and Oakhill, 1996) however; there is a variety of underlying reasons for such deficit (e.g., garden-variety, see, Nation and Snowling, 1998). Children might have strengths in one skill but deficits in others; they might be already skilled meta-cognitive readers and interruptions might disrupt their comprehension; they might as well have weaknesses in all three skills above described. We propose that any effective systems must be designed to provide a theoretically motivated context for learning and must have the ability to adapt to the user’s profile. The results presented here are in coherence with our claims and future work should be able to clarify some of the open questions stated in the present paper.

## Author Contributions

EG and GM developed the study concept, the experimental designed, prepared the materials and collected the data. GM implemented the website used for data collection. EG analyzed the data, interpreted the results and wrote the manuscript.

## Conflict of Interest Statement

The authors declare that the research was conducted in the absence of any commercial or financial relationships that could be construed as a potential conflict of interest.

## Footnotes

1 Only one participant in the sample manifested he was familiar with one of the stories he read. No other student mentioned having previously read or heard the stories.

2 R-code: glmer (accuracy ∼ condition : group + condition + group + (0 + condition | participant) + (0 + group | participant) + (0 + condition: group | participant) + (1 | participant), data, family = binomial).
