# Supplementary material for: A-Book: A Feedback-Based Adaptive System to Enhance Meta-Cognitive Skills during Reading
Source: Front Hum Neurosci. 2017 Mar 13;11:98. doi: 10.3389/fnhum.2017.00098 (PMC5346546; doi:10.3389/fnhum.2017.00098)
Supplement: Supplementary file 1 [file Data_Sheet_1.docx]

**APPENDIX**

Questions sample: 45 question from one of the stories presented to readers, 15 per meta-cognitive skill; inference-making, comprehension-monitoring and text structure-knowledge questions.

**Inference-making questions:**

1. Are angels aware of their children?

2. Do angels love children who behave well more than those who don’t?

3. Is Simon a very curious child?

4. Was the angel nervous?

5. Did Simon feel rejected for behaving well?

6. Was Simon's angel unconcerned with his godson?

7. Did the angels have permission to appear in front of their godchildren?

8. Do angels die together with their godchildren?

9. Were there many cases similar to Simon's?

10. Didn’t the angel feel like giving a signal to his godson?

11. Did Leonardo da Vinci see the silhouette of his angel just for an instant?

12. Did Simon's angel share the solution he found with the other angels?

13. Did Simon's angel copy the idea of Leonardo da Vinci's angel?

14. Did Simon think that the figure of milk and chocolate was his angel?

15. Had Simon made many drawings after seeing the figure of an angel?

**Comprehension-monitoring questions:**

1. Did you realize that the angels gather in the house of Simon?

2. Did you realize that Simon is not the godson of any angel yet?

3. Did you realize that the mother allowed him to say the name of the angel?

4. Did you notice that Simon did not change anything after talking to his mother?

5. Did you realize that Simon meant to be behaving worse day after day?

6. Did you realize that the angel crossed the coast of sparks?

7. Did you notice that the angel's wings flashed when he felt discouraged?

8. Did you realize that St. Francis and St. Teresa also had angels?

9. Did you realize that the other angel was small and had wings with a turquoise blue touch?

10. Did you realize that the rays of the sun illuminated the figure of the wall?

11. Did you notice that the cousins told Leonardo da Vinci that he was crazy?

12. Did you notice that the other angels were perplexed?

13. Did you realize that the child wanted to pour milk on his breakfast tray?

14. Did you notice that Simon started to paint?

15. Did you realize that the child in the end improved and felt happy?

**Text structure-knowledge questions:**

1. Is this the beginning of the story?

2. Is this story about to end?

3. Are we still seeing new characters appearing?

4. Do we already know what the end of the story is?

5. Are we still at the beginning of the story?

6. Did the story end?

7. Do we know where the story takes place?

8. Is this the beginning of the story?

9. Is the story over already?

10. Did we just meet a new character?

11. Are we about to solve Simon's problem?

12. Is the story ending?

13. Are we reaching the end of the story?

14. Are we still meeting characters from the story?

15. Did the story end?
